# Enhanced Visible Light-Driven Photoelectrocatalytic Degradation of Paracetamol at a Ternary z-Scheme Heterojunction of Bi_2_WO_6_ with Carbon Nanoparticles and TiO_2_ Nanotube Arrays Electrode

**DOI:** 10.3390/nano12142467

**Published:** 2022-07-19

**Authors:** Nondumiso Mahhumane, Leskey M. Cele, Charles Muzenda, Oluchi V. Nkwachukwu, Babatunde A. Koiki, Omotayo A. Arotiba

**Affiliations:** 1 Department of Chemistry, Tshwane University of Technology, Pretoria 0001, South Africa; nondumiso.mahhumane@gmail.com; 2 Department of Chemical Sciences, University of Johannesburg, Johannesburg 2028, South Africa; simbacharlee@gmail.com (C.M.); oluviv2000@yahoo.com (O.V.N.); bakoiki@gmail.com (B.A.K.); 3 Centre for Nanomaterials Science Research, University of Johannesburg, Johannesburg 2028, South Africa

**Keywords:** photoelectrocatalytic degradation, paracetamol, z-scheme heterojunction, bismuth tungstate, carbon nanoparticle, titania nanotube array, water treatment

## Abstract

In this study, a ternary z-scheme heterojunction of Bi_2_WO_6_ with carbon nanoparticles and TiO_2_ nanotube arrays was used to remove paracetamol from water by photoelectrocatalysis. The materials and z-scheme electrode were characterised using X-ray diffraction (XRD), field emission scanning electron microscopy (FESEM), energy dispersive X-ray spectroscopy (EDS), EDS mapping, ultraviolet diffuse reflection spectroscopy (UV-DRS), photocurrent measurement, electrochemical impedance spectroscopy (EIS), uv-vis spectroscopy and total organic carbon measurement (TOC). The effect of parameters such as current density and pH were studied. At optimal conditions, the electrode was applied for photoelectrocatalytic degradation of paracetamol, which gave a degradation efficiency of 84% within 180 min. The total organic carbon removal percentage obtained when using this electrode was 72%. Scavenger studies revealed that the holes played a crucial role during the photoelectrocatalytic degradation of paracetamol. The electrode showed high stability and reusability therefore suggesting that the z-scheme Bi_2_WO_6_-CNP-TiO_2_ nanotube arrays electrode is an efficient photoanode for the degradation of pharmaceuticals in wastewater.

## 1. Introduction

Paracetamol, also known as acetaminophen, is one the most prescribed drugs used for the treatment of pain, aches and fevers [1,2,3,4]. The discharge of paracetamol into aqueous environment can reach a toxic level that is harmful to humans and aquatic life [5]. Paracetamol reaches water bodies through the disposal of effluents, discharge of faeces/urine or through hospital sewage [6]. Due to the difficulty in the removal of paracetamol from water during treatment, advanced oxidation processes have been used for its removal in water instead of conventional methods [1,7]. Photo-fenton, sonolysis and H_2_O_2_/UV are extensively used advanced oxidation processes for the removal of paracetamol in water and wastewater [8,9]. Amongst other different types of AOPs, photoelectrocatalysis is gaining popularity due to its simplicity, low maintenance, positive environmental impact, recycling and reusability [10]. This method involves the use of a semiconductor immobilized on an electrochemical substrate [11,12,13]. Researchers have reported several different photoelectrocatalytic systems including single semiconductor systems. The problem with the single semiconductor systems for the degradation of paracetamol is that it cannot possess wide absorption and strong redox abilities simultaneously [14,15]. For instance, Bi_2_WO_6_ is a single semiconductor that has been used in the PEC degradation of organic pollutants [16]. Bi_2_WO_6_ is a typical Aurivillius oxide with a perovskite layered structure and a high surface area, which means it has sufficient active sites to promote photocatalysis [14,16]. Bi_2_WO_6_ also has a narrow band gap of 2.7 eV, which means it has strong light harvesting ability compared to TiO_2_. However, Bi_2_WO_6_ is limited by its low separation efficiency and its inability to fully utilize solar energy [15]. To overcome the challenge of low separation efficiency and improve the photocatalytic potency of Bi_2_WO_6_ (and most semiconductors in general), z-scheme photoanodes have been constructed via two semiconductors of dissimilar/similar band gap and one electron mediator or conductor. This system mimics the photosynthesis process which includes a two-step photoexcitation [14,15,17]. In a typical mechanism, electrons of photocatalyst I are recombined with holes in photocatalyst II via an electron mediator under light. This process inhibits the recombination of photo-induced charge carriers while they keep their redox properties [14,15,17,18].

In this study, bismuth tungstate and anodised TiO_2_ serve as photocatalyst I and II, respectively. TiO_2_ is the most widely studied semiconductor with properties such as low toxicity, cost effectiveness and excellent stability [19,20,21]. In recent years, researchers have synthesized a unique morphology of highly ordered TiO_2_ nanotube arrays (NTA). TiO_2_ NTA consists of a high surface area and unique cylindrical structure with improved electron transport efficiency compared to common TiO_2_ nanoparticles [22]. Amongst other different synthetic routes, anodisation of Ti sheets has been widely used, as it tends to give rise to a well-aligned structure with tuneable morphology and inner diameter. Anodised TiO_2_ are cost effective and provide better reusability compared to powdered TiO_2_ photocatalysts as they tend to disperse in solution during a reaction [22]. Unfortunately, TiO_2_ NTA only needs UV light for activation due to its wide band gap [22]. The formation of the z-scheme heterojunction with a narrow band gap semiconductor such as Bi_2_WO_6_, will solve this problem by tuning TiO_2_ NTA towards solar activation. To improve the conductivity of Bi_2_WO_6_ and TiO_2_ NTA, carbon nanoparticles (CNP) were used as an electron mediator. Nanomaterials have been incorporated into semiconductor structures as dopants or heterostructures for the enhancement of electrochemical, photocatalytic or photoelectrocatalytic properties, especially in the visible light [13,23,24].

Bi_2_WO_6_ has been applied in the photocatalytic degradation of organic pollutants and some of these reports used the z-scheme heterojunction approach. For example, Sharma et al. used a z-scheme of Bi_2_WO_6_ and titania for the degradation of antibiotics [14]. Hu et al. added porphyrin to Bi_2_WO_6_ for a z-scheme for photocatalytic application [15]. A recent review by Khedr et al. [25] highlights various photocatalytic applications of Bi_2_WO_6_. To the best of our knowledge, our work presents the photoelectrocatalytic application of a Bi_2_WO_6_ based photoanode for the first time. While TiO_2_ powder has been composited with Bi_2_WO_6_, this report used a novel route of anodized titanium sheet to form TiO_2_ NTA as a support for Bi_2_WO_6_ in the design of the z-scheme photoanode. The challenge of catalyst recovery, usually experienced in photocatalysis, is mitigated by the immobilization of the semiconductor on an electrode (photoanode) in photoelectrocatalysis, as in this case [10,26].

Bi_2_WO_6_ was coupled with CNP forming a Bi_2_WO_6_/CNP nanocomposite. The resulting Bi_2_WO_6_/CNP nanocomposite was immobilized on the surface of TiO_2_ NTA, giving rise to a novel ternary z-scheme photoelectrocatalyst (Bi_2_WO_6_/CNP/ TiO_2_ NTA) for the degradation of paracetamol under visible light. The extent of degradation was monitored by uv/vis spectroscopy and total organic carbon measurements.

## 2. Materials and Methods

### 2.1. Chemicals

Bismuth (III) nitrate pentahydrate, sodium tungsten oxide dehydrate, acetone, ethanol, nitric acid, orthophosphoric acid, ammonium fluoride and glucose were purchased from Sigma Aldrich, Johannesburg, South Africa and were used as received. All reagents were in analytical grade and were used without further purification.

### 2.2. Preparation of z-Scheme Bi_2_WO_6_/CNP/TiO_2_ NTA

Firstly, Bi_2_WO_6_ was synthesized by Sharma et al.’s 2020 method with slight modification, using the solvothermal method. A mass of 1.455 g of bismuth nitrate pentahydrate (Bi(NO_3_)_3_5H_2_O) was solubilized in 37.5 mL of ethylene glycol, instead of deionized water, and labelled as solution A. Next, 0.5 g of sodium tungstate dehydrate (Na_2_(WO_4_)2H_2_O) was dissolved in 12.5 mL of ethylene glycol and labelled as solution B. Solution B was introduced to solution A under sonication with dropwise addition. The resulting solution was stirred for an hour and then transferred into an autoclave for solvothermal treatment at 160 °C for 25 h. After the reaction, a brown precipitate was collected via centrifugation, washed with ethanol and water thoroughly and dried at 50 °C overnight.

The Bi_2_WO_6_-CNP composite was prepared by the dispersion of synthesized Bi_2_WO_6_ in absolute ethanol and sonicated for 50 min to obtain uniformity of suspension of the particles followed by the addition of 10 to 30% weight of the previously prepared CNP, using a method suggested by Tshwenya and Arotiba [27]. The mixture was further sonicated for 30 min. The mixture was placed in the oven to dry at 100 °C overnight for the complete evaporation of ethanol. The resulting composite was placed in the muffle furnace for 2 h at 500 °C.

A highly ordered TiO_2_ NTA was prepared using the approach reported by Koiki et al. [22]. Titanium sheets of 5 × 3 cm were used as the substrate for the fabrication of TiO_2_ NTA. Before the anodisation process, the sheets were sonicated in detergents acetone, ethanol, nitric acid and deionised water, respectively, and dried in the oven. The anodisation process was performed with a two-electrode electrochemical cell connected to a DC power source. A platinum foil was used as a counter electrode and was placed at a 2 cm distance from the titanium sheet anode. A potential of 25 V was applied to the electrochemical cell, which contained a mixture of 0.2 M H_3_PO_4_ and 0.3 M NH_4_F as the electrolyte for 2 h while being stirred magnetically. Thereafter, the prepared electrode was rinsed with deionised water to eliminate blocked ions from its surface and subsequently annealed for 2 h with a heating and cooling rate of 10 °C/min. The Bi_2_WO_6_-CNP composite was immobilized on the anodized TiO_2_ NTA via a simple deposition where 10% wt of nafion was used as a binder. The electrode was then placed in the oven and left to dry overnight.

### 2.3. Characterisation

The crystalline properties of the fabricated powders were measured by X-ray diffraction (XRD, D8 Advance, Bruker, Germany). The morphology and structure were observed by scanning electron microscopy (SEM, Supra55, Zeiss, Germany) and transmission electron microscopy (TEM, JEM-2100, JEOL, Tokyo, Japan), and the element constitution was detected by energy-disperse X-ray spectroscopy (EDS). The Brunauer–Emmett–Teller (BET) surface areas of the as-prepared materials were measured using a surface area and porosity analyzer (Quadrasorb EVO, Quantachrome, Florida, USA) by nitrogen adsorption–desorption. The optical properties of various photocatalysts were examined by UV–Vis diffuse reflectance spectra (UV–Vis DRS, UH4150, Hitachi, Japan).

### 2.4. Electrochemical and Photoelectrochemical Characterisation

The electrochemical and photoelectrical measurements was conducted on Autolab PGSTAT204 (Barendrecht, Netherland) potentiostat/galvonstat with a three-electrode configuration. The fabricated electrode, platinum foil and Ag/AgCl (3.0 M KCl) were used as working electrodes, counter electrode and reference electrode, respectively. An Oriel LCS-100 W solar simulator with UV cut-off (λ less than 400 nm) was used as the light source for PEC experiment. The prepared electrode was positioned vertically facing the incident light of the simulator and the distance between the photoelectrochemical cell and the light source was kept constant at 10 cm. Photocurrent measurements and linear sweep voltammetry was carried out in a 0.1 M Na_2_SO_4_ solution. Cyclic voltammetry was carried out in a 5 mM solution of [Fe(CN)_6_]^3−/4−^ (prepared in a 0.1 M KCl). For the degradation experiments, paracetamol solutions were prepared in 0.1 M Na_2_SO_4_ supporting electrode. Electrochemical degradation experiments were carried out in the dark. An aliquot of the solution was collected from the reactor at certain time intervals using a disposal syringe. The concentration decay and degradation pattern of the analyte was observed using UV-visible spectrophotometer and the total organic removal was recorded using TOC. The effects of applied potential on the removal efficiency were investigated.

**Figure 1:**
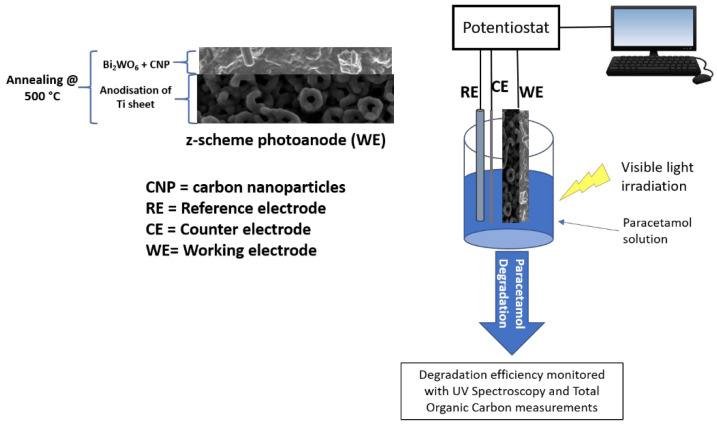
Schematic diagram of prepared Bi_2_WO_6_-CNP-TiO_2_ NTA z-scheme photoanode used for photoelectrocatalytic degradation of paracetamol in a three-electrode cell.

Figure 1 presents the schematics of the photoanode and the photoelectrochemical set-up for the degradation of paracetamol.

## 3. Results

### 3.1. Structural and Morphological Characterisation

**Figure 2:**
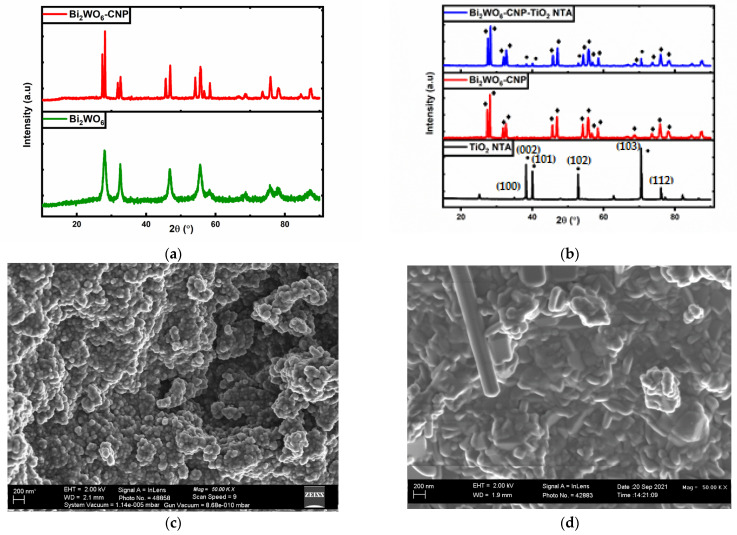

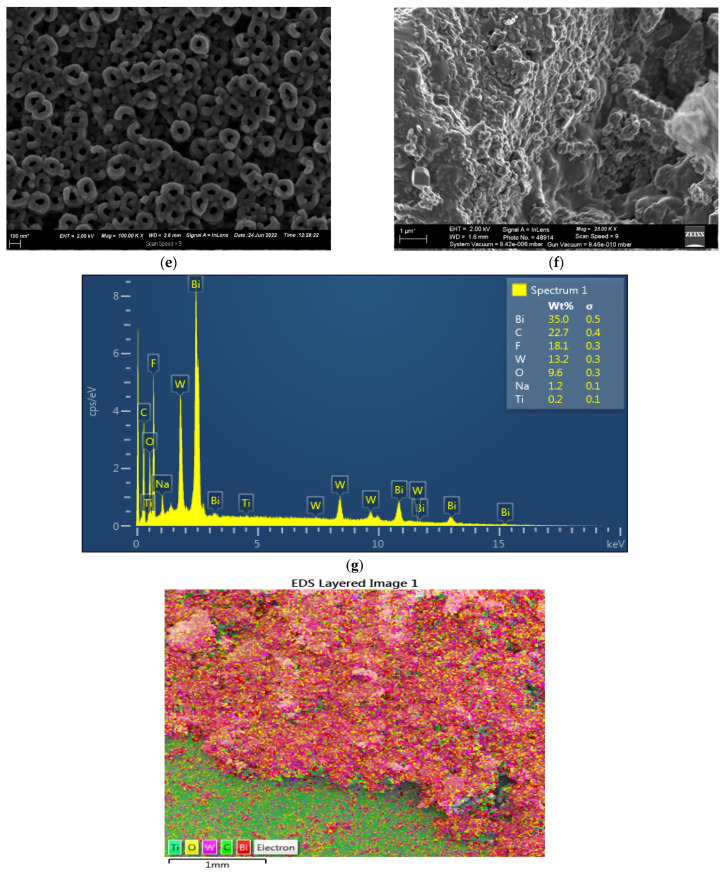

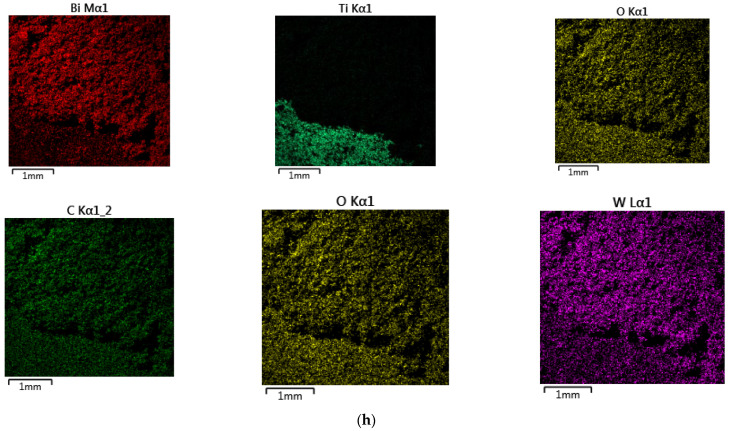
XRD patterns of (**a**) Bi_2_WO_6_ and Bi_2_WO_6_-CNP, (**b**) TiO_2_ NTA and Bi_2_WO_6_-CNP-TiO_2_ NTA. FESEM images of (**c**) Bi_2_WO_6_; (**d**) Bi_2_WO_6_-CNP; (**e**) TiO_2_ NTA; (**f**) Bi_2_WO_6_-CNP-TiO_2_ NTA; (**g**) EDS spectrum of Bi_2_WO_6_-CNP-TiO_2_ NTA and (**h**) EDS elemental mapping of Bi_2_WO_6_-CNP-TiO_2_ NTA electrode.

The crystal structure of the synthesized Bi_2_WO_6_ and Bi_2_WO_6_-CNP composite was characterised by XRD as indicated in Figure 2a. The Bi_2_WO_6_ diffraction peaks at 2θ = 28.34°, 32.88°, 47.06°, 55.99°, 58.59°, 68.92°, 75.97° and 78.33° are attributed to the orthorhombic phase of the Bi_2_WO_6_ and are in agreement with JCPDS card no. 39-0256 (Sharma et al., 2020). However, after the addition of CNP on the surface of Bi_2_WO_6_, new diffraction peaks were observed. The sharpest peak found at 28.7° may be an influence of highly disordered carbon atoms with amorphous nature, as seen in most carbon materials. Figure 2b presents the crystal structure of synthesized TiO_2_ NTA and Bi_2_WO_6_-CNP-TiO_2_ NTA. During the synthesis of TiO_2_ NTA, it is important to control parameters such as the composition of the electrolyte, anodization potential and annealing temperature to achieve highly ordered tubes [22]. For instance, the use of a viscous electrolyte may lead to an excessive supply of oxygen ions than needed for oxidation [22]. The use of ethylene glycol may provide a slow anodic oxidation process and formation of glycolaldehyde and glycolic acid, making the donation of oxygen challenging [22]. Hence, the use of H_3_PO_4_ is advantageous, since it serves as a buffer and prevents rapid etching during nanotube growth [22]. Accurate anodization potential must be used, as high potential can damage the surface structure, resulting in disordered spongy porous tubes. The annealing temperature is also important as it gives rise to anatase and rutile form. At temperatures higher than 500 °C, the anatase and rutile form begins to combine and at 800 °C, they completely disappear [22]. As seen in Figure 2b the synthesized TiO_2_ NTA shows diffraction peaks at 2θ = 35.1°, 38.4°, 40.2°, 53°, 70.6° and 76.2°, corresponding to (100), (002), (101), (102), (103) and (112) diffraction plane of Ti metal, respectively, and are in agreement with JCPDS card no. 04-004-7631. The TiO_2_ NTA only showed anatase peaks at 2θ = 25.3°, 48.1°, 62.7°, and 75.1°, corresponding to (101), (200), (204) and (205) in agreement with JCPDS card no 01-075-2546. This suggested that pure TiO_2_ anatase was formed. After the deposition of Bi_2_WO_6_-CNP on the surface of TiO_2_, the peaks belonging to Bi_2_WO_6_-CNP and TiO_2_ NTA were observed on the XRD pattern of Bi_2_WO_6_-CNP- TiO_2_ NTA, confirming the successful synthesis of the ternary composite. The crystallite size of Bi_2_WO_6_ and Bi_2_WO_6_-CNP was calculated from Debye-Scherrer’s formula, given by

D = 0.94 λ/β cos θ

where D is the average crystallite size, λ is the wavelength of X-ray radiation, β is the broadening of the diffraction line assessed at full width half maximum value (FWHM) and θ is the Bragg’s angle. The crystallite size of Bi_2_WO_6_ and Bi_2_WO_6_-CNP are 9.36 nm and 5.32 nm, respectively. The decrease in crystallite size shows that the Bi_2_WO_6_-CNP has an increased surface area than the pure Bi_2_WO_6_. BET analysis was carried out to further confirm the increase in surface area where Bi_2_WO_6_ gave a surface area of 6.3214 m^2^/g and Bi_2_WO_6_-CNP gave a surface area of 15.0235 m^2^ g^−1^.

The surface morphology of the prepared Bi_2_WO_6_, Bi_2_WO_6_-CNP, TiO_2_ NTA and Bi_2_WO_6_-CNP-TiO_2_ NTA was investigated using FESEM, as seen in Figure 2c–f. The pure Bi_2_WO_6_ shows spherical aggregate morphology (Figure 2c). However, with Bi_2_WO_6_-CNP the morphology of the aggregate changed to rod-like structures, as seen in Figure 2d. Figure 2e shows pristine TiO_2_ NTA with highly ordered and well-oriented tubes, with an estimated diameter of 98 nm and wall thickness of 18 nm around them. After deposition, the agglomerated rod-like structures of Bi_2_WO_6_-CNP cover the surface of the TiO_2_ NTA as seen in Figure 2f. However, the TiO_2_ holes can be seen within the Bi_2_WO_6_-CNP, indicating that both materials are present on the electrode. The EDS spectrum in Figure 2g further confirms the elemental composition of Bi_2_WO_6_-CNP-TiO_2_ NTA with the following elements present in different proportions and quantities: Bi, Ti, W, C, and O. Other elements seen on the spectrum are due to the binder used for deposition. The EDS mapping analysis in Figure 2h validates the existence and even distribution of the elements Bi, Ti, W, C, and O, making up the Bi_2_WO_6_-CNP-TiO_2_ NTA electrode.

### 3.2. Optical Properties

**Figure 3:**
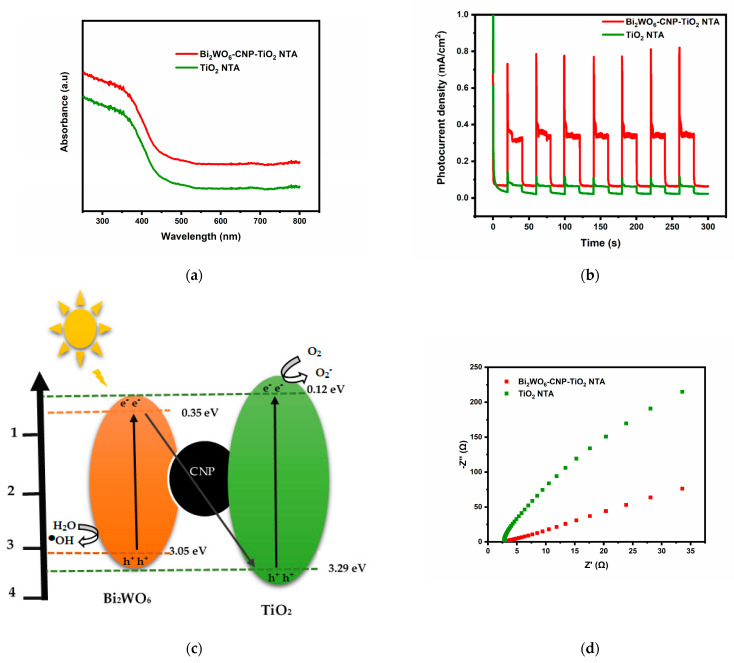
(**a**) UV-Visible diffuse reflectance spectra TiO_2_ NTA Bi_2_WO_6_-CNP- TiO_2_ NTA (**b**) photocurrent responses of TiO_2_ NTA and Bi_2_WO_6_-CNP- TiO_2_ NTA in 0.1 M Na_2_SO_4_ (**c**) schematic diagram of Z-scheme mechanism of Bi_2_WO_6_-CNP-TiO_2_ NTA electrode (**d**) Nyquist plot TiO_2_ NTA and Bi_2_WO_6_-CNP- TiO_2_ NTA photoanodes in 5 mM [Fe(CN)_6_]^3−/4−^ in 0.1 M KCl (pH 7).

UV-vis diffuse reflectance spectroscopy shows that the Bi_2_WO_6_-CNP-TiO_2_ NTA electrode has higher absorbance than the TiO_2_-NTA electrode in the visible region of 400 to 800 nm (Figure 3a). This suggests that the formation of the z-scheme heterojunction has enhanced visible light activity.

### 3.3. Electrochemical Studies

Figure 3b shows the chronoamperographs of pristine TiO_2_ NTA and Bi_2_WO_6_-CNP- TiO_2_ NTA under solar light illumination at 2 V bias potential using 0.1 M KCl solution. The two electrodes showed photocurrent responses (improved current in the presence of light than in the dark). However, the Bi_2_WO_6_-CNP-TiO_2_ NTA electrode exhibits a more marked photocurrent response with a higher current density of 0.789 mA cm^−2^ when compared to bare TiO_2_ NTA with a current density of 0.172 mA cm^−2^. The photocurrent density of Bi_2_WO_6_-CNP- TiO_2_ NTA is about 4.6 times higher than the TiO_2_ NTA. The increase could result from the z-scheme of heterojunction between Bi_2_WO_6_, CNP and TiO_2_ NTA, giving rise to an improved separation of electron hole pairs (Figure 3c). To further confirm the improved performance of the prepared electrodes an experiment was conducted using electrochemical impedance spectroscopy. The experiment was performed in an electrolytic solution of 5 mM [Fe(CN)_6_]^3−/4−^ in 0.1 M KCl at pH 7 with external application of +0.2 V. Figure 3d shows the Nyquist plot of the fabricated photoanodes consisting of a semicircle, which represents the charge transfer process happening at the solution-electrode interface. The diameter of the semicircle represents the charge transfer resistance (Rct) at the interface of the electrode. Lower Rct values indicate a better charge transfer efficiency. The Rct of TiO_2_ NTA and Bi_2_WO_6_-CNP- TiO_2_ NTA are 92.14 Ω and 31.32 Ω, respectively. This suggested that the fabrication of the z-scheme heterojunction has improved the charge mobility of the electrode and lowered the rate of instantaneous recombination of electron-hole pairs [11].

### 3.4. Photoelectrochemical Studies

**Figure 4:**
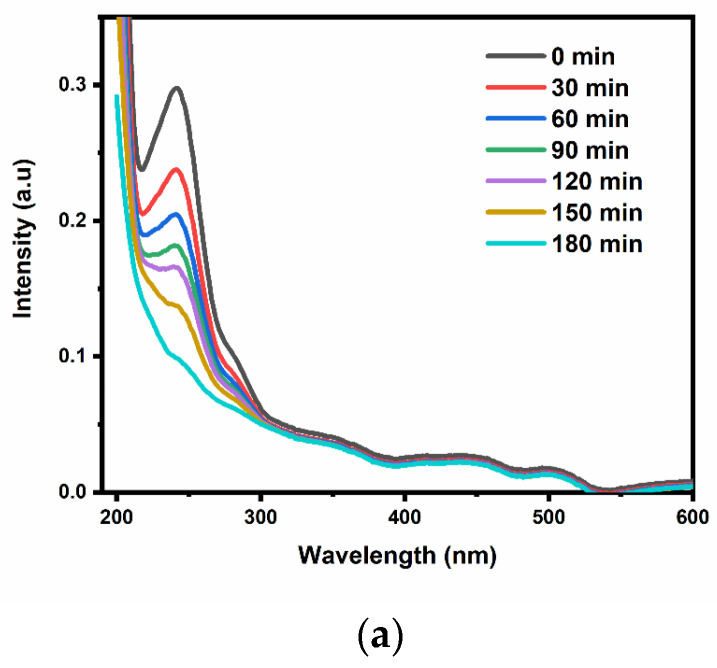

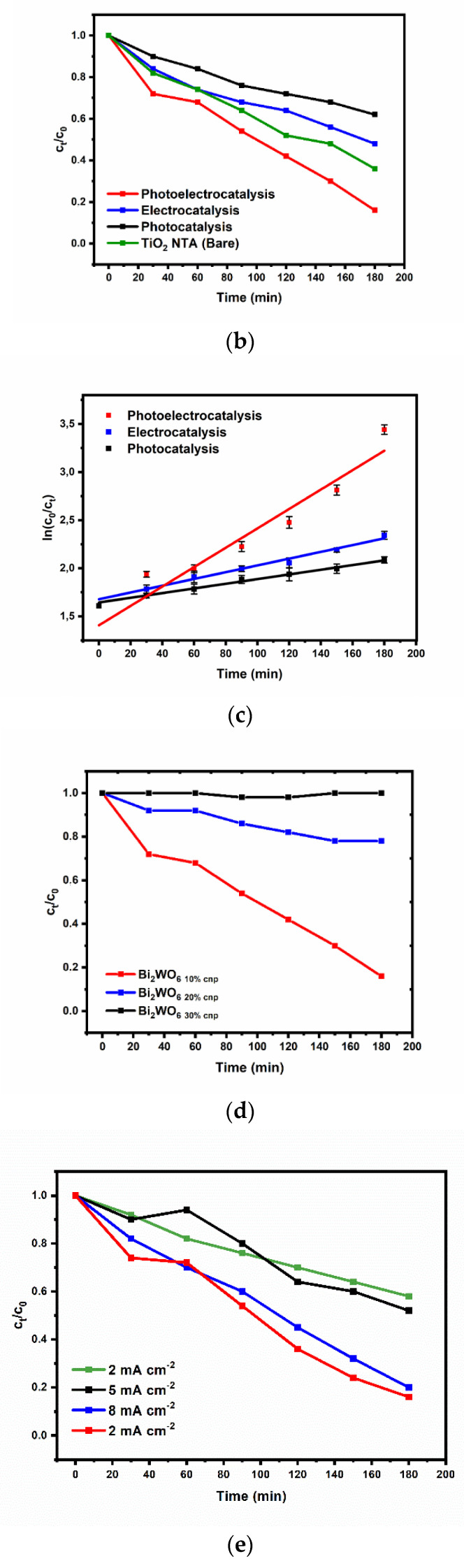

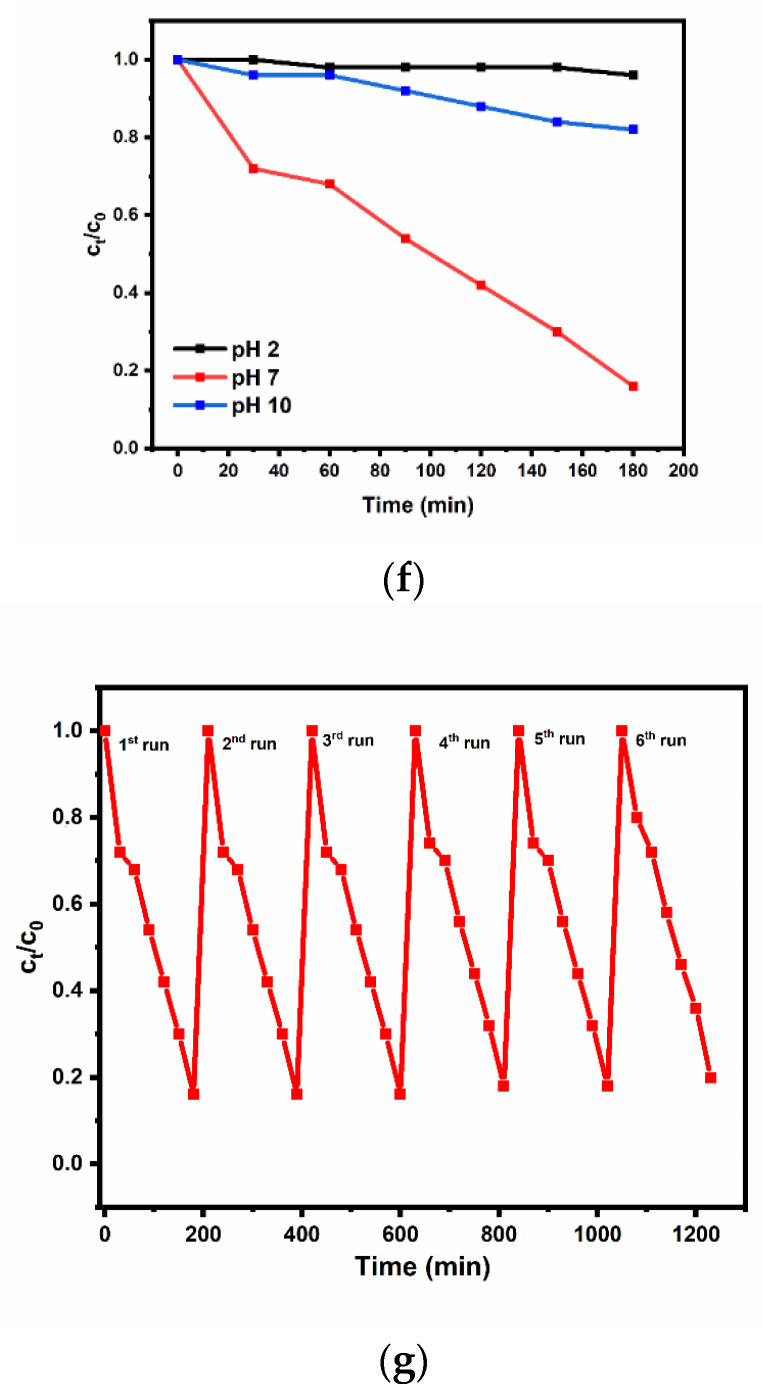
(**a**) UV-Vis spectra of PEC degradation of paracetamol; (**b**) normalised concentration decay versus time plot for photocatalytic, electrocatalytic and photoelectrocatalytic degradation of paracetamol on of Bi_2_WO_6_-CNP–TiO_2_ NTA electrode and (**c**) corresponding kinetics plots; (**d**) normalised concentration decay versus time plot for PEC degradation of Paracetamol on of Bi_2_WO_6_-CNP–TiO_2_ NTA electrode. (**e**) Effects of current density and (**f**) pH on the degradation of paracetamol. (**g**) Reusability cycle experiments for the degradation of paracetamol on of Bi_2_WO_6_-CNP–TiO_2_ NTA electrode.

The photoelectrocatalytic performance of the prepared electrodes was evaluated by applying it in a solar reactor for the degradation of paracetamol with an initial concentration of 5 mg L^−1^. The degradation was accomplished with an applied current of 10 mA cm^−2^, pH 7 and irradiation with solar light. UV-visible spectrophotometry was used to monitor the degradation efficiency of the electrode at a wavelength of 243.0 nm. Figure 4a shows the decrease in the intensity of the peak at 243.0 nm as the reaction time increases, representing the reduction in the concentration of the pharmaceutical. A degradation efficiency of 84% was achieved after 3 h when using the z-scheme electrode for photoelectrocatalysis, as presented in Figure 4b. When the Bi_2_WO_6_-CNP–TiO_2_ NTA photoanode was applied for electrochemical oxidation of the paracetamol (that is without the stimulation of solar light), the degradation efficiency decreased to 57% (Figure 4b). In photocatalysis, where only the solar light was applied, a 42% degradation efficiency was recorded (Figure 4b). Photoelectrocatalytic (PEC) degradation of the paracetamol at the TiO_2_ NTA only (that is without Bi_2_WO_6_) yielded 62%. These degradation percentages show that the formation of a z-scheme enhanced the PEC process. The degradation of paracetamol using the z-scheme electrode was further confirmed by the percentage of total organic carbon removal (TOC) which was 72, 30 and 22% for PEC, electrochemical oxidation (EC) and photocatalysis (PC), respectively. EC is the same setup as PEC but without irradiation with visible light, while PC is without the application of potential. This observation confirms that the synergy between solar energy and applied bias potential is crucial for breaking down the organic pollutant. Consequently, the solar energy promotes the generation of electron–hole pairs on the surface of the electrode, while the biased potential helps with the transfer of photo-excited electrons, which reduces the recombination of electron-hole pairs.

The kinetic of paracetamol degradation for each process was investigated using Langmuir-Hinshelwood pseudo first order kinetic equation. The degradation rate constant using the z-scheme electrode for PEC, EC and PC were 0.00987 min^−1^, 0.00352 min^−1^ and 0.00243 min^−1^, respectively. This reveals that the degradation of paracetamol was faster when the z-scheme electrode was applied in photoelectrocatalysis, as shown in Figure 3c. Figure 4d depicts the effect of different amounts of CNP to Bi_2_WO_6_ on the fabrication of Bi_2_WO_6_-CNP–TiO_2_ NTA photoanode. The results clearly indicate that above 10% wt of CNP, the degradation of paracetamol is suppressed, therefore it is important to keep the amount of CNP minimal for maximum PEC degradation efficiency. Since 10% gave the highest degradation efficiency, it was used for the preparation of the Bi_2_WO_6_-CNP–TiO_2_ NTA electrode. The effect of parameters such as current density and pH were investigated as shown in Figure 4e,f, respectively. The degradation efficiency increased with an increase in current density ranging from 5 mA cm^−2^ to 10 mA cm^−2^ (Figure 4e). This shows that the production of hydroxyl radicals depends on the applied current density. However, current densities should be kept at the optimum to prevent electrode leaching and the production of scavenging radicals. A high current density gives rise to oxygen evolution, which prevents the generation of hydroxyl radicals, which can suppress the degradation efficiency [12].

The surface charge of the catalyst, drug molecule, radicals and solvent molecules affects the photoelectrocatalytic reaction process under different pH conditions (Sharma et al., 2020). According to previous studies, the point of zero charge for Bi_2_WO_6_ and TiO_2_ are 5.5 and 6.2, respectively (Sharma et al., 2020). Therefore, at an acidic pH value, the surface of the catalyst becomes positively charged. As presented in Figure 3f under acidic conditions (pH 2), a degradation efficiency of 4% was obtained. It was also reported that paracetamol is in its protonated form under acidic conditions [28], hence the result obtained could be due to the repulsion between the surface of the catalyst and the drug molecule. At pH 10, a degradation efficiency of 18% was obtained. In alkaline conditions, more hydroxyl radicals are formed; however, paracetamol is in its deprotonated form [28], thus the repulsion between the negatively charged solution and the drug molecule could minimize the degradation efficiency. A maximum degradation efficiency of 84% was obtained at pH 7 where paracetamol is in its neutral form.

An ideal photoanode is projected to be stable and reusable. The stability and reusability of the prepared of Bi_2_WO_6_-CNP–TiO_2_ NTA photoanode was investigated by using the same electrode six times; after each cycle the electrode was gently rinsed with deionized water and air dried. As shown in Figure 4g, the degradation efficiency decreased by only 2% after the sixth cycle. This suggests a good stability and reusability of photoanode for PEC degradation.

### 3.5. Plausible Mechanism for Paracetamol Degradation

The position of the conduction band and valence band of Bi_2_WO_6_ and TiO_2_ was determined using the calculations from UV-DRS studies and the following equations:

E_VB_ = X_e_ − E_e_ + 0.5E_g_ (1)

E_CB_= E_VB_ − E_g_ (2)

where E_CB_, E_VB_, X_e_, E_e_ and E_g_ are the conduction band of the semiconductor, valence band of the semiconductor, the absolute electronegativity of the semiconductor, energy of free electrons on the hydrogen scale (4.5 eV) and the band gap energy of the semiconductor, respectively. Therefore, E_VB_ and E_CB_ of Bi_2_WO_6_ are 3.05 eV and 0.35 eV, while E_VB_ and E_CB_ for TiO_2_ are 3.285 eV and 0.12 eV, respectively. Based on the approximate bandgap energy values of Bi_2_WO_6_ and TiO_2_ NTA a multiple charge transfer mechanism is followed for the degradation of paracetamol. When the prepared Bi_2_WO_6_-CNP–TiO_2_ NTA is irradiated by solar light, Bi_2_WO_6_ and TiO_2_ NTA undergo excitation, where electrons migrate from the VB to the CB, leaving positively charged holes on the VB and electron (negative charge) on the CB. However, the electrons on the CB of Bi_2_WO_6_ migrate through the CNP to the holes on the VB of TiO_2_ NTA. The electron transportation that occurs through the carbon nanoparticles enhances the separation of electrons and holes of CB of TiO_2_ NTA and VB of Bi_2_WO_6_, respectively. The separated electrons on the CB of TiO_2_ NTA and the separated holes on the VB of Bi_2_WO_6_ make TiO_2_ NTA electron-rich and Bi_2_WO_6_ hole-rich. The carbon nanoparticles on the surface of Bi_2_WO_6_ act as a charge transfer bridge, where electrons from Bi_2_WO_6_ migrate to holes in TiO_2_ NTA. The increased number of electrons on TiO_2_ NTA react with adsorbed O_2_ to form superoxide radicals. The holes in the VB of Bi_2_WO_6_ can oxidize H_2_O to form hydroxyl radicals, which also participates in the degradation of paracetamol.

**Figure 5:**
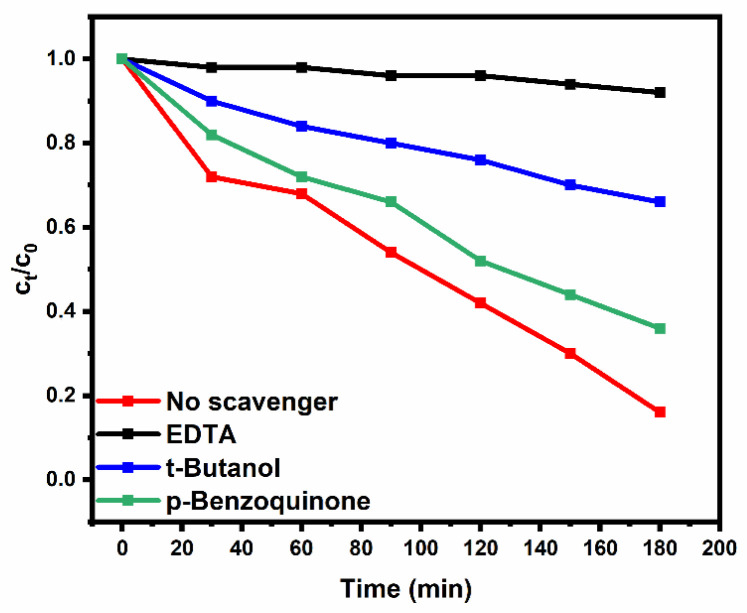
Scavenger studies of the photoelectrocatalytic degradation of Paracetamol on Bi_2_WO_6_-CNP-TiO_2_ NTA.

The role of the active species for photoelectrocatalytic degradation of paracetamol was investigated by conducting a radical suppressing experiment (Figure 5). The holes, hydroxyl radical and superoxides were suppressed using ethylenediaminetetraacetate (EDTA), t-butanol and p-benzoquinone, respectively. When EDTA was added to the solution, the degradation efficiency dropped significantly to 8%. On the other hand, when t-butanol and p-benzoquinone were added in the solution the degradation efficiency was 34 and 64%, respectively. The results indicate that the hole and hydroxyl radicals play a major role in the degradation of paracetamol, while superoxides play a minor role. The photoelectrocatalytic degradation of paracetamol can be projected to follow Equations (3)–(7).

hv + Bi_2_WO_6_-CNP–TiO_2_ NTA → Bi_2_WO_6_-CNP–TiO_2_ NTA (h_VB_^+^ + e_CB_^−^) (3)

e_CB_^−^ + O_2_ → ^●^O_2_^−^ (4)

h_VB_^+^ + H_2_O → ^●^OH + H^+^ (5)

^●^OH + Paracetamol → CO_2_ + H_2_O (6)

^●^O_2_^−^ + Paracetamol → CO_2_ + H_2_O (7)

h_VB_^+^ + Paracetamol → CO_2_ + H_2_O (8)

## 4. Conclusions

A visible light driven ternary z-scheme Bi_2_WO_6_-CNP-TiO_2_ NTA heterojunction photoanode was successfully prepared for photoelectrocatalytic application of the water treatment process. FESEM images showed that the catalyst was formed by an agglomerated rod-like structure of Bi_2_WO_6_-CNP and highly ordered TiO_2_ NTA, while EDS mapping supported the formation of the catalyst by showing all the elements present. The formation of the z-scheme heterojunction enhanced the visible light activity of the semiconductor heterojunctions. The formation of the z-scheme also improved the photocurrent response to about 4.6 times higher and charge carrier separation. When the z-scheme heterojunction photoanode was applied for PEC degradation of paracetamol, a degradation efficiency of 84% was obtained within 180 min. Scavenger studies revealed that the holes played a major role in the degradation process. The electrode showed excellent stability and reusability, hence the reported photoanode shows great potential for the photoelectrochemical application of organic pollutants.

## Data Availability

Not applicable.
